# Systems and Synthetic Biology Approaches to Engineer Fungi for Fine Chemical Production

**DOI:** 10.3389/fbioe.2018.00117

**Published:** 2018-10-03

**Authors:** Leonardo Martins-Santana, Luisa C. Nora, Ananda Sanches-Medeiros, Gabriel L. Lovate, Murilo H. A. Cassiano, Rafael Silva-Rocha

**Affiliations:** Systems and Synthetic Biology Laboratory, Cell and Molecular Biology Department, Ribeirão Preto Medical School, São Paulo University (FMRP-USP), Ribeirão Preto, Brazil

**Keywords:** synthetic biology, yeast, filamentous fungi, genetic engineering, bioinformatics, biotechnology

## Abstract

Since the advent of systems and synthetic biology, many studies have sought to harness microbes as cell factories through genetic and metabolic engineering approaches. Yeast and filamentous fungi have been successfully harnessed to produce fine and high value-added chemical products. In this review, we present some of the most promising advances from recent years in the use of fungi for this purpose, focusing on the manipulation of fungal strains using systems and synthetic biology tools to improve metabolic flow and the flow of secondary metabolites by pathway redesign. We also review the roles of bioinformatics analysis and predictions in synthetic circuits, highlighting *in silico* systemic approaches to improve the efficiency of synthetic modules.

## Overview

Systems biology has risen as a relevant approach to encompass the phenomena that occur concomitantly in determined environments. Since its advent, research was modeled after a global perception of biological systems operation. Systems biology presents scientific possibilities to understand processes as a whole and to integrate new data and new analytical tools that are intended to solve biological problems by integrating parts and modules. Specifically, systems biology offers us the possibility to study the combination of individual parts which can play different roles in the behavior of the biological system depending on the context.

On the order hand, synthetic biology refers to the use of rational approaches that provide us with the possibility to study a unique part from a module or a system in a minimalist manner to understand how the creation of nonexistent parts could enable us to engineer biological circuits, for the engineering of microbes as cell factories. These approaches seek to address the growing industrial demand for biotechnological processes that manufacture products in the required scale in an economically efficient manner. Many efforts in the genetics and metabolic engineering fields have explored the use of viable fungal cells to generate fine chemicals.

Synthetic biology plays a central role in these efforts by means of innovating the use of the components of biological circuits, pathway, or processes and ultimately of systematically harnessing these modules to achieve the goals of industrial scale production. The advent of the bioinformatics era has allowed the redesigning of living information in the cell environment. This has significantly contributed to the development of new strategies based on systems biology approaches, mainly computational and rational ones. Since all these advances in single parts of a system and integrative modules direct efforts toward a common goal, systems and synthetic biology permit the deeper exploration and elucidation of cellular mechanisms, mainly the genetic and metabolic engineering of fungal cells in an integrative way.

Here, we review and discuss the most promising recent advances in this field, focusing on the use of single module, genetic and metabolic designs to engineer filamentous fungi and yeasts for industrial biotechnological processes. We describe some of the most promising industrially relevant tools for this purpose. Also, a list of significant studies that have used synthetic biology approaches to engineer fungi is provided in Table 1.

**Table 1:** Synthetic biology approaches for fine chemical production with filamentous fungi and yeast as cell biofactories.

**Approach**	**Organism**	**Strategy**	**References**
Promoter library construction	*P. pastoris*	Synthetic promoter engineering	Ata et al., 2017
Transcriptional circuit sensitive to xylose	*A. gossypii*	Synthetic promoter engineering	Hector and Mertens, 2017
Cellulase optimization promoter dynamics	*T. reesei*	Synthetic promoter engineering	Kiesenhofer et al., 2017
Improvement of promoter strength	*S. cerevisiae*	Intronic sequences in promoters	Hoshida et al., 2017
Evaluation of terminators function improvement	*S. cerevisiae*	Nucleosome occupancy arrangement predictions	Morse et al., 2017
Galactaric acid production improvement	*A. niger*	CRISPR/Cas9	Kuivanen et al., 2016
Yeast genome engineering	*P. pastoris*	Optimized codons for Cas9 and RNA polymerases promoter sequences	Weninger et al., 2016
Synthetic biopathway control	*S. cerevisiae*	CRISPR/dCas9	Jensen et al., 2017
Improvement of production and tolerance to ethanol	*S. cerevisiae*	Polymerase engineering	Qiu and Jiang, 2017
Expression of cellulase genes through a copper responsive promoter	*T. reesei*	RNA interference	Wang et al., 2018
Stable segregation of vectors	*S. stipiti*	Episomal vector optimization	Cao et al., 2017
Tolerant acetic acid mutant yeast	*S. cerevisiae*	Direct evolution approach	González-Ramos et al., 2016
Responsiveness to low-pH conditions	*S. cerevisiae*	Synthetic promoter engineering	Rajkumar et al., 2016
Redirected carbon flux from acetyl-CoA to ß-carotene production	*Y. lipolytica*	Fine-tuning expression of synthetic genes	Gao et al., 2017
Production of terpenes production	*R. toruloides*	Codon optimization of biosynthetic enzyme coding genes	Yaegashi et al., 2017
Isobutanol production	*S. cerevisiae*	Mitochondrial compartmentalization pathway	Park et al., 2016
Controlling accumulation of free fatty acids	*S. cerevisiae*	Dynamic regulatory circuits	Teixeira et al., 2017
Production of alkaloids	*S. cerevisiae*	Proof-of-concept synthetic circuit	Galanie et al., 2015

## Molecular engineering tools

### Synthetic promoters

Transcriptional control redesign is a general term encompassing processes used to engineer fungi that have been the most intensively studied ones. Many single modules that are involved are pivotal to drive heterologous protein expression. Advances in synthetic promoter design have significantly contributed to the creation of engineered strains. In this sense, Ata et al. (2017) recently reported the construction of a synthetic promoter library based on the glyceraldehyde-3-phosphate dehydrogenase *(G3PDH)* promoter in *Pichia pastoris*, a well-known methylotrophic yeast model. The study adopted a robust strategy of simultaneous deletions and duplications of transcription factor binding sites (TFBSs) to control expression of transcription factor (TF) genes to understand the transcriptional dynamics in these cells. This is a promising approach to unravel the mechanisms of transcriptional dynamics that could be potentially expanded to further investigations of the transcription logic in yeast, with the ultimate goal of robust and improved strain engineering.

**Figure 1:**
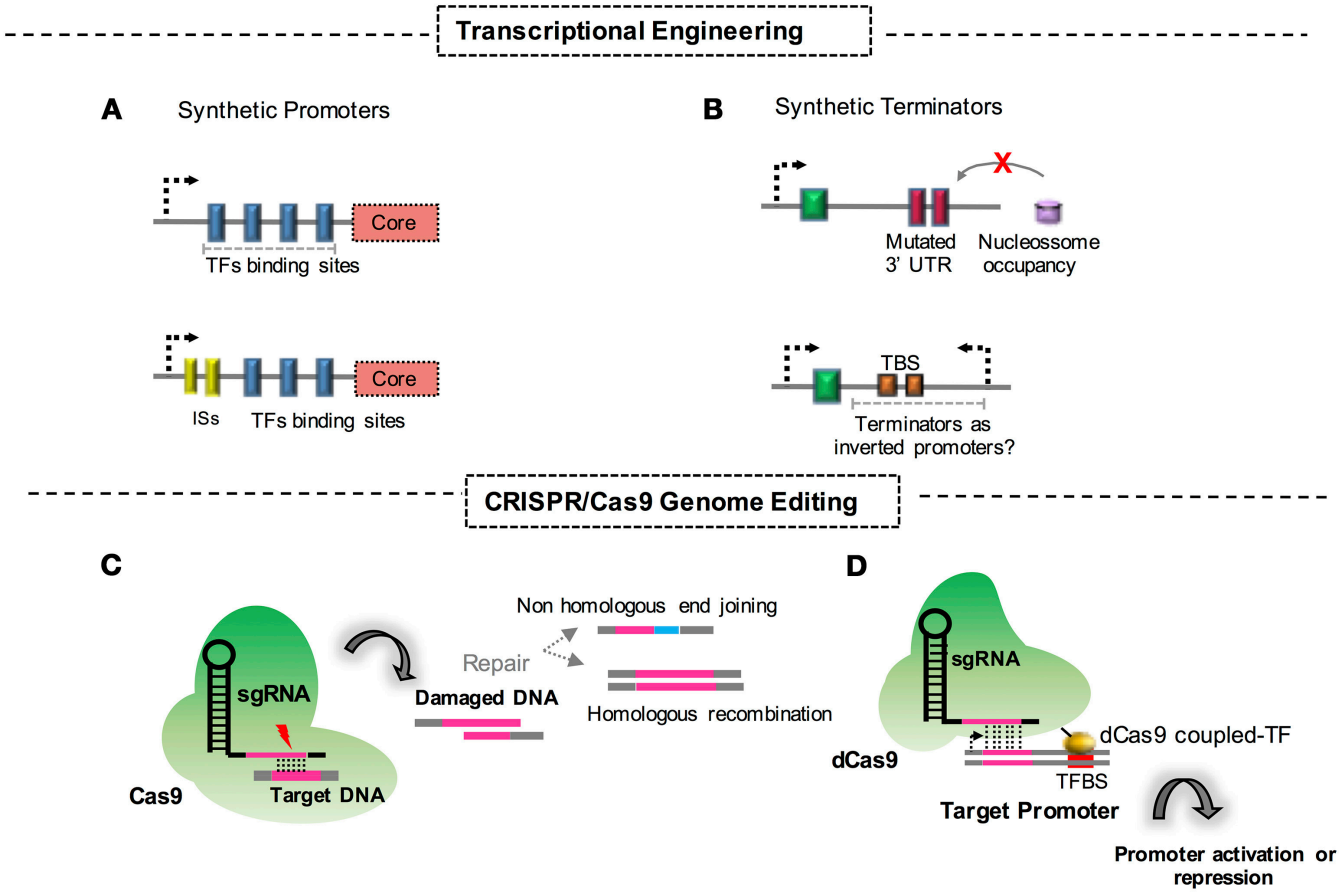
Synthetic biology approaches and strategies for engineering fungi. **(A)** Construction of synthetic promoters through replacement of protein binding domains at the corresponding DNA sequence. ISs, intronic sequences. **(B)** Synthetic terminators constructed through mutational approaches of DNA untranslated regions. TBS, terminator basic sequences for transcriptional termination; UTR, untranslated region; **(C)** Genome editing through CRISPR/Cas9 strategies and its respective DNA double strand repair mechanism. **(D)** CRISPR/dCas9 fused to a TF system aimed at activation or repression of a promoter.

This strategy allows the creation of promoter variants with different strengths in the presence or in the absence of critical regulators. It relies on the premise that these dynamics directly influence the transcriptional response and the regulation logic. Approaches like these are the most common and robust tools for understanding the dynamics of regulation in yeast cells. The design of a set of promoters that rely on different architectural compositions is crucial for the systemic understanding of regulation dynamics and is a convenient application for more detailed studies to engineer strains for industrial purposes (Figure 1A). Although the potential of the approach in yeast engineering is clear, further studies are required for its successful application in the engineering of filamentous fungi.

Another elegant strategy to engineer fungal promoters consists of a series of molecular designs to obtain hybrid promoters using bacterial modules. Standard tools from bacteria could be easily implemented to investigate transcriptional behavior in eukaryotic cells. This approach was proposed in a recent study that reported the construction of synthetic promoters based on the *Ashbya gossypii* translation elongation factor (TEF) promoter architecture (Hector and Mertens, 2017). In this study, the authors used the negative regulator *xylR* from the gram-negative bacterium, *Caulobacter crescentus*, to construct a xylose-sensitive transcriptional circuit. For this, the authors replaced specific TF recognition regions of the promoter using the bacterial DNA, which involved the modification of upstream and downstream TATA flanking regions. This strategy proved to be relevant for the engineering of yeast for use in fine chemical production, mainly in biomass conversion processes (Hector and Mertens, 2017).

The replacement and new insertions of (TFBSs) are elegant ways to engineer promoters, as are combinatorial fusions of regulatory elements creating novel, promoter architectures. Both approaches could optimize transcription in fungal cells. One of the greatest benefits of this approach is the possibility to supply the industrial demand for metabolites or fine chemicals, which is currently limited by extreme chemical conditions, such as low pH. A systematic and expansive molecular strategy to overcome the limits of transcriptional regulation is needed to increase the level of production of high-value added products. To this end, a construct based on the core architecture of *G3PDH* gene promoter complementary with the upstream activating sequence of the guanylate kinase gene promoter was explored to guarantee transcriptional responsiveness to pH oscillations in *Candida glycerinogenes*. The resultant promoter was used for the expression of an industrially valuable enzyme, xylose dehydrogenase, in low pH conditions, heralding the approach as a relevant strategy for metabolic bioprocess in such conditions (Ji et al., 2017). The same logic could be applied for other purposes in processes that take place in a basic pH environment or in the presence of various stress conditions.

Although promoter engineering is a promising approach, new engineering technologies and strategies are required especially for filamentous fungi. Some studies have explored the optimization of transcriptional regulation systems to understand how the disposition and repetition of TFBSs in DNA can influence the dynamics of regulation. A recent study reported the construction of engineered promoters from two wild type *Trichoderma reesei* promoters, the most utilized fungus for the production of cellulases (Kiesenhofer et al., 2017). The findings of this work have indicated the relevance of the number of repeated TFBSs for transcription regulation and that the disposition of the recognition sites plays a major role in the final response of the system. This strategy could be further explored to engineer fungi with redesigned metabolic flow and to investigate regulatory complexity in these organisms.

#### Effects of introns in transcriptional regulation

Synthetic promoters can also be engineered with the use of portions of a gene. Insertion of introns upstream of specific promoter sequences is a typical example of this form of engineering (Figure 1A). Molecular recognition by the transcriptional cell machinery has been recently used to promote a transcriptional response following the integration of intron sequences upstream of a promoter sequence. One example is the insertion of introns in promoter regions of relevant genes involved in lipid biosynthesis in *Rhodosporidium toruloides* (Liu et al., 2016). With a vector containing a luciferase gene under the transcriptional control of the promoters in the study, the authors successfully harnessed the intronic sequences to modulate the promoter strength. As a result, the promoter activity of synthetic promoters was 3-fold higher when compared with some wild type sequences. The improvement in the strength of the modified promoters of perilipin/lipid droplet protein 1 gene, acetyl-CoA carboxylase gene, and fatty acid synthase subunit β gene, makes this strategy feasible for the engineering of yeast promoters for industrial applications (Liu et al., 2016).

Another recent study investigated the influence of introns in promoter sequences in *Saccharomyces cerevisiae*. In this case, the authors demonstrated that the presence of 5′ untranslated regions (UTRs) of intronic sequences in the promoter of genes encoding 40S ribosomal proteins (RPs) increased the strength of the promoter. The luciferase activity was 17-fold higher when an intronic promoter was used to drive expression when compared with the strong wild type TDH3 promoter in *S. cerevisiae* (Hoshida et al., 2017). Furthermore, when the RPS25A intronic promoter and TDH3 promoter were merged, the luciferase activity increased, approximately, by 50-fold when compared with the control of the TDH3 promoter alone (Hoshida et al., 2017). Further studies on these combinations will alleviate the remaining bottlenecks due to intron off-effects and expand the strategy to the engineering of filamentous fungi.

### Synthetic terminators

Terminator sequences constitute an important part of molecular regulatory modules in a circuit. These sequences are pivotal in the final steps of transcription and are required for the complete and successful generation of the mRNA machinery, owing to their involvement in mRNA stability, and even as insulators in genetic circuits (Curran et al., 2013; Geisberg et al., 2014; Song et al., 2016). In synthetic biology, terminators are significant modules that guarantee precise and controlled regulation. These functions have heightened the research focus on terminator sequences. Decades ago, scientists had already focused their efforts on creating synthetic and minimal terminators to study how these elements could influence transcription and mRNA half-life (Guo and Sherman, 1996). In the intervening decades, further studies have demonstrated the use of engineered terminators as suitable tools for synthetic biology applications. In this context, the direct relationship of a 3′ UTR of *S. cerevisiae* dityrosine-deficient 1 (DIT1) terminator to increased protein expression was described as a process mediated by NAB6p and PAP1p *trans*-acting RNA-binding proteins. This relationship was demonstrated by the analysis of mutations of the DIT1 terminator sequence. The mutations that were introduced improved the terminator activity by 500% when compared with an internal terminator used as the control (Ito et al., 2016). This approach is relevant for the study of post-transcriptional control mechanisms, and it demonstrates the influence of terminators on mRNA stability and the levels of protein production *in vivo*.

With evidence from multiple studies that the half-life of mRNA could be affected by terminator sequences, the idea that molecular arrangements in DNA strands may reflect that the distribution of terminators has gained acceptance in the scientific community. Recently, Morse et al. (2017) described that the function of *S. cerevisiae* terminators could be modulated based on the predicted nucleosome occupancy arrangements. The study exploited the fluorescence emission that occurred under the control of designated promoters to demonstrate that the engineered minimal terminators of the *cyc1* and *adh1* genes may positively influence protein production via decreased nucleosome affinity (Morse et al., 2017). The authors also explored the hypothesis that terminators behave as antisense promoters in the generation of noncoding RNA molecules. To investigate this, the authors reversed the orientation of the yECitrine fluorescence reporter gene to evaluate a possible transcriptional control of the synthetic terminator in question. However, the results that were obtained failed to confirm the hypothesis of a terminator acting as an antisense promoter in this case. The authors suggested alternative mechanisms for the absence of differential trends of the described synthetic terminators in the study, and the data supporting these alternatives could be found in the literature (Morse et al., 2017).

Fewer studies have addressed the activity and influence of terminators on transcriptional and post-transcriptional regulation when compared to the studies describing promoter engineering. Therefore, further investigations are necessary to gain a full understanding of the properties of terminators. However, the present knowledge indicates that the intrinsic properties of terminators make them a suitable tool for synthetic biology approaches that will be useful for industrial applications in the not too distant future (Figure 1B).

### CRISPR/Cas9 tools

Clustered regularly interspaced short palindromic repeats (CRISPR) tools for the engineering of microbes as cell factories are an efficient and reliable strategy in synthetic biology. Fungal cells, even filamentous species, have already been modified for their successful use in industrial bioprocesses. The CRISPR/CRISPR-associated protein 9 (Cas9) technique, basically, consists of using a Cas9 endonuclease-guided RNA to target a DNA sequence, which allows the precisely targeted disruption of this sequence and the activation of repair mechanisms in cells. This strategy fits a chimeric single guide RNA (sgRNA) and a Cas9 into a unique and easily manipulatable plasmid vector. Since the appearance of CRISPR/Cas9, numerous efforts have aimed to engineer fungal cells for industrial purposes (Figure 1C). Recent advances deserve special attention and are addressed in this review. For instance, the filamentous fungi *Aspergillus niger* was engineered with CRISPR/Cas9 to create a strain capable of producing galactaric acid from galacturonic acid, a representative molecule present in pectin fibers. In this study, authors bring to light the fact that galactaric acid can be used as a carbon source for *A. niger*. To create a strain capable of retaining this molecule, the CRISP/Cas9 system was used to efficiently disrupt the metabolic pathway for this compound (Kuivanen et al., 2016). This is a remarkable study in the field, considering the fact that fungi from the *Aspergillus* genus have a considerable genetic capability to produce enzymes for hydrolysis of biomass content such as pectin, as well as established raw material for industrial applications.

A major advantage of using the CRISPR/Cas9 system to edit genomes is the system's plasticity for repairing double-strand DNA breaks. Led by sgRNA, Cas9 cleaves the target DNA, which activates a repair mechanism in eukaryotic cells. Therefore, the nonhomologous end joining system of repair is considered an error-prone method for fixing broken sequences and may generate disruptions in these sequences by deleting or incorporating small DNA molecules to the site of damage. This type of repair system is a suitable alternative to generate knock down strains or to disrupt an open reading frame (ORF) of the gene of interest. Additionally, the repair promoted by homologous recombination provides an error-free system through the incorporation of a faithful donor DNA sequence, which is a valuable strategy when the goal is to replace a sequence of interest.

The CRISPR/Cas9 system can be applied for both yeast and filamentous fungi strains, even with the considerable genetic and morphological differences between the two groups. Despite the limitations of recombination, this technology has been used to create a series of functional constructs of *P. pastoris* in a precise and efficient manner (Weninger et al., 2016). In this case, the authors tested combinations of single modules in a robust system featuring optimized codons for Cas9, as well as optimized RNA polymerase II and III promoter sequences (Weninger et al., 2016). This approach is promising for future applications in yeast genome editing and is anticipated to allow the creation of efficient standardized strategies for the engineering of filamentous fungi. In addition to this case, recent studies have demonstrated the application of this technology to *Yarrowia lipolytica* (Schwartz et al., 2017), *A. oryaze* (Katayama et al., 2016), *A. fumigatus* (Zhang et al., 2016), *C. albicans* (Shapiro et al., 2017)*, Penicillium chrysogenum* (Pohl et al., 2016), for the hyper-expression of cellulase using *Myceliophthora* species (Liu et al., 2017), and even for the creation of broad spectrum promoters (Yang et al., 2017). Therefore, it becomes clear that this tool will be increasingly important for the generation of new strains with the improvement of the functions that are required for biotechnological applications.

### CRISPR/Cas9 and transcription regulation

As addressed before, breakthroughs using the CRISPR/Cas9 technique have been achieved even with the inherent limitations of the method, such as the occurrence of low recombination rates and off-targets in the genome. One of the most important improvements generated recently has been the coupling of a transcription factor to a mutated/dead Cas9, which is generally termed dCas9 (Figure 1D). This technique leads to a CRISPR/Cas9 system but with the power of inhibiting transcription when Cas9 blocks the sites for RNA polymerase anchoring in promoters, reducing the number of transcripts in a system. Additionally, molecular approaches have also been employed to fuse transcription regulatory domains of proteins to promote the enhancement or repression of transcription.

Singular approaches have also been developed that combine a dCas9 and engineered genomic RNA, as discussed by Jensen et al. (2017). In this case, the authors described the creation of different systems of transcriptional response that were modulated using dCas9 to control synthetic pathways in *S. cerevisiae*. They also proposed that differently predicted genomic RNAs could influence the reprogramming regulation in different promoters of the yeast (Jensen et al., 2017). The results indicated that the occurrence of multiplex reprogramming strategies to engineer yeast for the production of triacylglycerols (TAGs) and isoprenoid compounds, but further studies are required for the expansion and optimization of industrial applications.

### Molecular tools for transcriptional regulation

A wide array of possible molecular fungal engineering tools is available in the literature. Here, we briefly review the more notable and the newest synthetic biology approaches to engineer fungi for fine chemical production.

#### RNA interference (RNAi)

**Figure 2:**
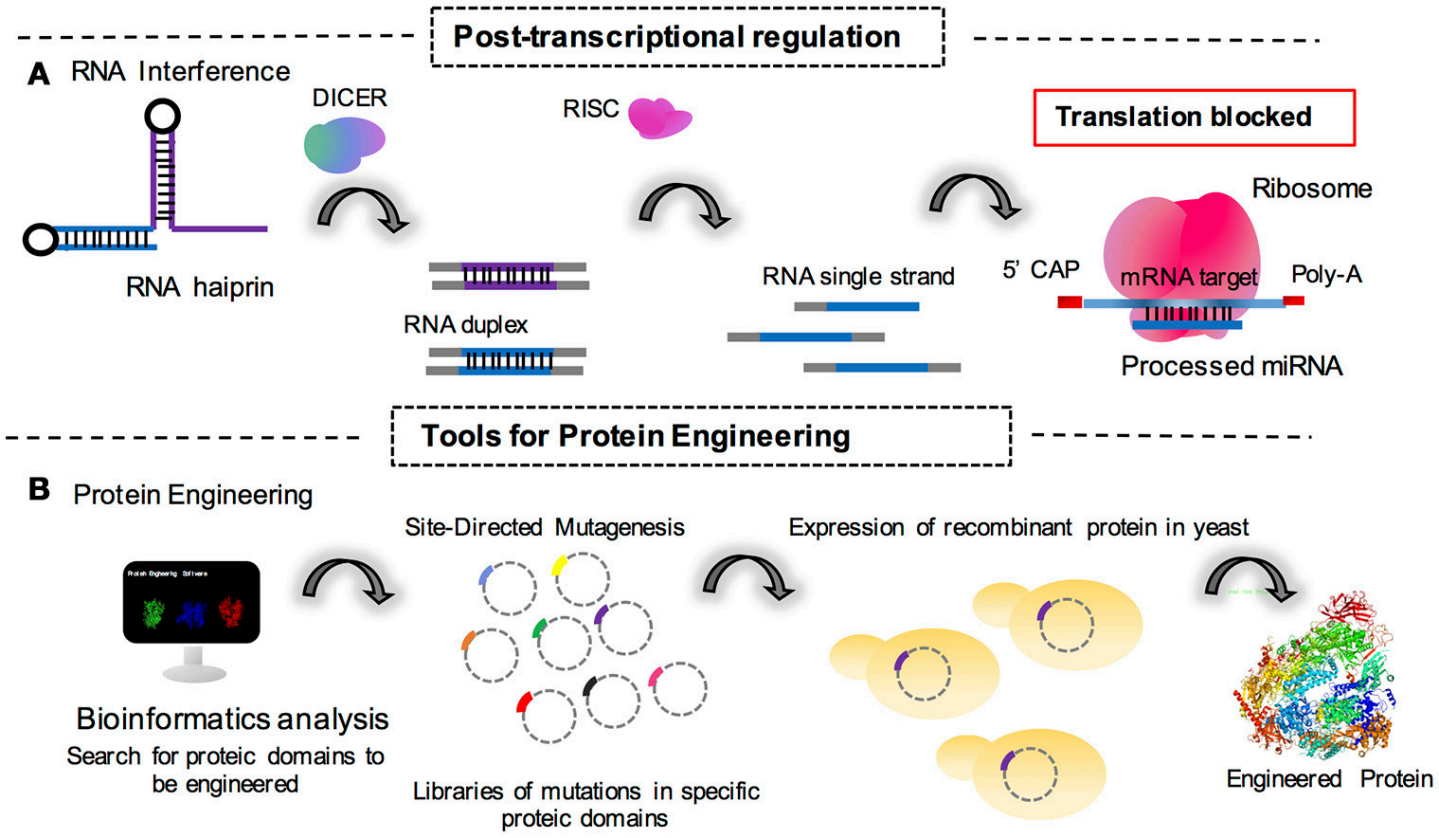
Synthetic tools for the fungal genetics and metabolic engineering cited in this review. **(A)** Post-transcriptional regulation mediated by translation block for the action of cell machinery processed microRNAs (miRNAs) as interference RNA molecules. **(B)** Engineering of proteins through mutational approaches mediated by vector amplification containing the mutated gene sequence in a library of DNA mutated sequences for protein domains. Monomeric structure of *S. cerevisiae* RNA polymerase is available in the 5LMX Protein DataBank access code; Torreira et al. (2017).

Post-transcriptional control is also a point of study for synthetic biologists to understand how mRNA or non-coding RNAs may behave as knockdown agents for the regulation of protein expression (Figure 2A). In this sense, Wang et al. (2018) reported the use of RNAi in *T. reesei* to successfully create a system for the inhibition and derepression of cellulase genes under transcriptional control of the copper responsive *tcu1* promoter. In this system, the absence of copper in the medium promoted the transcription of a hairpin RNA for the genes of interest. The presence of copper in the medium also repressed transcription in a system of toggle modulation for post-transcriptional control (Wang et al., 2018).

#### Polymerase engineering

Protein engineering is a reliable strategy for industrial synthetic biology (Figure 2B). With this method, it is possible to create a library of mutant protein domains in order to improve the catalytical potential of any given enzyme to be further applied in industrial processes. Recently, it was reported that mutations on subunit Rpb7 of RNA polymerase II (generated by error-prone PCR) in *S. cerevisiae* improves the production and tolerance to ethanol in bioprocesses (Qiu and Jiang, 2017). This study highlighted a simple but very efficient technique for strain engineering. This was the first study to describe the engineering of eukaryotic polymerases to modulate transcriptional responses in yeast (Qiu and Jiang, 2017). The study additionally highlights the elegance of simple approaches for synthetic biology and paves the way for more investigations that will make it possible to engineer the same procedure in filamentous fungi for industrial purposes.

#### Episomal optimization

Cao et al. (2017) used fine engineering to improve episomal expression. The authors identified minimal regions in centromeres that were capable of promoting the stable segregation of vectors in *Scheffersomyces stipitis*, an unconventional yeast that the authors studied for the production of shikimate pathway-derived compounds owing to the native capacity of the yeast to metabolize xylose. This is a remarkable milestone in the field of synthetic biology, both for the identification of centromere functional minimal regions and for the successful application of CRISPR/Cas9 for an unconventional yeast species, paving the way for novel strategies to engineer novel strains for industrial purposes (Cao et al., 2017).

#### Orthogonality by engineered TFs

Engineered TFs can be used to improve the transcriptional response in living cells. A recent and innovative study used engineered TFs with yeast, virus, and plant activation domains that were fused to a nuclear localization signal and a full-length TF or its DNA binding domain. In this case, the authors noticed that transcription of a yeast-enhanced green fluorescent protein reporter gene under the control of a yeast promoter was differentially modulated by each domain (Naseri et al., 2017). The authors also discussed the influence of the number of binding sites embedded in such promoters and noticed that some artificial TFs derived from *Arabidopsis thaliana* could also modulate the transcription of the fluorescence pattern in the presence/absence of an inducer (Naseri et al., 2017). This approach expands the possibilities for the future application of synthetic biology in other eukaryotic cells.

## Metabolic engineering

Metabolic engineering is an ongoing challenge in the microbial production of fine chemicals from sustainable biomass. For many years, metabolic engineers had to rely on random mutagenesis and screening of strains, meaning that any adverse characteristics could not be easily detected (Campbell et al., 2017). However, with the advent of synthetic biology, it has become possible to circumvent most of these constraints and improve bioprocesses, allowing the enhanced manipulation of carbon flux in fungal strains for the production of chemicalas, food additives, pharmaceutical products, and other molecules of interest (Wang et al., 2016). A relevant example of metabolic engineering applied to fungal strains is the production of fine chemicals through plant biomass hydrolysis. Plant biomass is mainly composed of lignin, cellulose, and hemicellulose. These compounds, frequently present in agro-industrial wastes, can be hydrolyzed to fermentable sugars thought the action of a number of specific enzymes. For this purpose, fungal strains can be engineered to produce those enzymes that can break specific bonds in such polymers and generate sugars, such as glucose and xylose, which can be directed to the production of ethanol, butanol, fatty acids, and aromatic compounds.

The base of biotechnological production from biomass is cellulose and hemicellulose. When hydrolyzed, they generate pentoses and hexoses, which can be converted to generate biosustainable commodities (Guerriero et al., 2016; Gupta et al., 2016). There are two different approaches to obtain these products from fungi. One approach is for the production of non-oleaginous biofuels and chemicals and the other is for the production of oleaginous compounds. For the non-oleaginous bioproducts, synthetic biology approaches focus on the glycolysis of C5 and C6 sugars and their further conversion to the product of interest, in which the pyruvate flux is redirected to alternative pathways (Chen and Nielsen, 2016). Examples include lactic acid, succinic acid, *cis*-muconic acid, and ethanol, which are molecules important for polymer production and for applications in the cosmetic, food, chemical, and biofuel industries (for further details, please refer Chen and Nielsen, 2016).

Metabolic engineering for the production of oleaginous compounds is focuse on the central carbon metabolism, with the aim of increasing the molecular levels of acetyl-CoA, malonyl-CoA, and acyl-CoA. This generates precursors for the production of fatty acids that are used for the manufacture of detergents, lubricants, biodiesel, plastics, and coatings (Marella et al., 2018). Although some fungal species, such as *S. cerevisiae*, fail to produce high levels of cytosolic acetyl-CoA and require metabolic manipulation to circumvent this absence, there are species (*Y. lipolytica*, for example) that naturally produce higher rates of this molecule. Therefore, strategies to manipulate both species have been reported (Jin et al., 2015; Marella et al., 2018) and subsequently reviewed in detail. Nevertheless, studies have reported the use of *S. cerevisiae* as a chassis for the production of fatty acids, in which biomass is used as a carbon source for animal feed supplementation through the addition of acetyl-CoA carboxylase and thioesterase genes from *Corynebacterium glutamicum* (You et al., 2017). Another application is the production of oil with reduced viscosity (composed by acetyl-TAGs) with the introduction of a diacylglycerol acetyltransferase from *Euonymus alatus* (Tran et al., 2017). In addition, Wei et al. (2017a) successfully engineered *S. cerevisiae* with six cocoa genes to produce a cocoa butter-like (CBL) lipid.

Many studies concerning waste biomass utilization have focused primarily on hemicellulose as the main carbon source. However, lignin, a rich aromatic resource and an underutilized product from biomass hydrolysis, is another lignocellulosic feedstock that could be successfully employed for biochemical production. A major challenge in the utilization of lignin is the variety of aromatic compounds that are present in this material. Thus, engineering the metabolism of fungi is required to depolymerize and convert complex aromatic macromolecules of lignin to a utilizable resource (Beckham et al., 2016; Xie et al., 2016). In this sense, Yaegashi et al. (2017) described the capacity of *R. toruloides* to consume aromatic compounds as lignin components and suggested that this organism has the metabolic potential to convert depolymerized lignin into lignocellulosic sugars. Moreover, Mahan et al. (2017) and Liu Z. H. et al. (2018) described the ways to improve lignin utilization by *Rhodococcus opacus* for the production of oleaginous compounds, with the use of pretreatments and fermentation that favors lignin hydrolysis. It would be interesting to apply these techniques in fungal engineering with the goal of lignin utilization.

### Construction of resistant strains

One of the first steps in the creation of cell factories to produce fine chemicals from biomass is to overcome metabolite inhibition. Several metabolites generated by hydrolysis and further assimilation of substrates can inhibit the metabolism of the microorganisms and even block the production of the desired substances (Zhang et al., 2015). Especially, regarding lignocellulosic biomass, even more challenges exist because the biomass is a complex of carbon backbones whose hydrolysis could generate furan derivatives, phenolic compounds, and weak organic acids that could behave as fermentation inhibitors. Furthermore, inhibitor substances could also be generated as intermediates of the reaction or as products (Ling et al., 2014). This highlights the need to create resistant strains as an indispensable strategy to improve the entire biotechnological process.

Another problem during the biomass fermentation is the acidification of the growth medium due to the acidic pretreatment of the biomass (Fletcher et al., 2017). In this regard, Chen et al. (2016) examined transcriptional responses from *S. cerevisiae* during fermentation stress and acidic environments, given that lignocellulosic material often undergoes acidic pretreatment. By identifying genes that are induced under acidic condition, the authors reported that the simultaneous overexpression of a protein related to acid resistance (Sfp1) and a protein related to the general stress response (Whi2) increased yeast performance and ethanol productivity, even in highly acidic media.

As another example, González-Ramos et al. (2016) used a direct evolution approach to identify *S. cerevisiae* mutants that tolerated different concentrations of acetic acid. Additionally, whole-genome sequencing of the tolerant strains was performed to identify the mutations contributing to this phenotype, with the aim of identifying genes worthy of further study with regard to acidic resistance. Notwithstanding this, Ma et al. (2015) developed an *S. cerevisiae* mutant strain tolerant to acetic acid by transforming it with a synthetic zinc finger protein transcription factor (ZFP-TF) library, and they identified genes that could be related to this acetic acid resistance phenotype. The authors also observed that the glucose consumption rates and ethanol productivity in media containing acetic acid were increased in the mutants when compared with the wild type. In another study, Rajkumar et al. (2016) engineered a yeast synthetic promoter that was responsive to low pH. For this, they improved the already existing responsiveness to the low pH YGP1 promoter, altering its TFBSs and selecting the best responsive system. As a result, the engineered promoter was associated with a 10-fold improvement in the transcription rate when compared with a commonly utilized promoter. Therefore, the intersection between this synthetic promoter technique and the aforementioned direct evolution and engineering strategies could assist in the identification of a microbe that can strive in highly acidic conditions.

As mentioned before, the sensitivity to the product generated by a metabolic process is another bottleneck that needs to be circumvented for industrial purposes. For example, the production of fatty acids by *S. cerevisiae* leads to toxic effects that include membrane and mitochondria disruption and oxidative stress. Besada-Lombana et al. (2017) engineered *S. cerevisiae* strains with increased tolerance to octanoic acid, whose presence compromises plasma membrane integrity. For this, the authors cloned a copy of the acetyl-CoA carboxylase gene with a mutation in S1157A to increase the concentration of oleic acid in the plasma membrane. The engineered strains displayed increased tolerance to octanoic acid, n-butanol, 2-propanol, and hexanoic acid molecules, indicating that this strategy can be a promising tool to improve tolerance to products from metabolic processes.

Seeking novel means for engineering fungal metabolism has always been a challenge, and emerging techniques are trying to explore the stress response in the organisms of interest to improve metabolic tolerance. In this sense, Li et al. (2017) investigated the ethanol stress response mechanism of *S. cerevisiae* by RNA-seq to understand how the cells can be manipulated to overcome ethanol sensitivity. In the landscape of unconventional organisms, this understanding could allow the exploitation of the yeast biodiversity to generate new tools for metabolic engineering of industrial domesticated strains. Mukherjee et al. (2017) have described some unconventional yeasts, such as *P. kudriavzevii* and *Wickerhamomyces anomalus*, which have an elevated tolerance to stress factors present in fermentation processes. Studies like this are extremely relevant to the development of new tools for the metabolic engineering of fungi, and they will contribute to the improvement of biomass usage and generation of high value-added products in the diverse scope of biotechnological processes.

## Compartmentalization of pathways and protein scaffolds

As proof that compartmentalization is important to optimize metabolic pathways, an efficient strategy to improve the use of acetyl-CoA focused on targeting the pathway proteins to the mitochondrial matrix. In this context, Yuan and Ching (2016) exploited the compartmentalization of acetyl-CoA utilization pathways by taking advantage of the subcellular metabolism of *S. cerevisiae* to avoid competing intermediates. They were able to produce amorphadiene using the mitochondrial acetyl-CoA pool by using a plasmid to overexpress amorphadiene synthase in the mitochondria. The production was considerably more efficient in this case (about 80% higher) than when the same enzyme was expressed in the cytosol, demonstrating that the mitochondrial matrix can be a desirable environment to redirect metabolic pathways. Furthermore, a pathway for isobutanol production was also improved by localizing biosynthetic enzymes to the mitochondria of *S. cerevisiae*. Park et al. (2016) increased the pool of mitochondrial pyruvate by overexpressing the subunits of the hetero-oligomeric mitochondrial pyruvate carrier. Higher titers of isobutanol production were evident in this case when compared with the wild type and to previous reports.

As another important example, DeLoache et al. (2016) proposed a redesign of the metabolic flux in yeast peroxisome. The authors engineered mutant yeast strains in which the peroxisomes became “synthetic organelles,” meaning they could receive heterologous proteins and control the influx of substrates. This study not only developed novel *in vivo* assays to test cargo import and to measure membrane permeability, but it also identified a modular tag for peroxisome localization. The findings showed that this methodology has the potential to redirect metabolic flux in yeast species, including *S. cerevisiae* and *P. pastoris*. For an extensive review on the localization of heterologous proteins to different yeast cell compartments, please refer Hammer and Avalos (2017).

Synthetic protein scaffolds, on the other hand, interact with the enzymes of natural pathways via peptide ligands, co-localizing them, and assembling them into organized clusters. This enables the direct transfer of substrates from one active site to another, which is termed substrate channeling (Wheeldon et al., 2016). In this sense, Wang and Yu (2012) used this concept to construct nine synthetic scaffold proteins to enhance the overall cascade catalysis of the resveratrol biosynthesis pathway in *S. cerevisiae*. Resveratrol is an antioxidant that is of interest to pharmaceutical companies. The authors successfully produced this compound using *p*-coumaric acid that was derived from lignin. The study revealed that the number of binding domains can affect the flux through the biosynthetic pathway.

In an even more sagacious study, Lin et al. (2017) expressed synthetic scaffold proteins from the ethyl acetate biosynthesis pathway in *S. cerevisiae* with a localization tag to lipid droplets, based on the idea that co-localization of enzymes would provide kinetic advantages, and balance the metabolic flux and substrate channeling. In fact, there was an increase in the metabolic rates due to the nanometer spacing between the co-localized enzymes, even in the presence of competing substrates. These findings indicated that compartmentalization along with organization and spatial distribution must be considered while developing tools to engineer fungal metabolism.

## Synthetic biology tools for rewiring carbon metabolism

The flow of metabolites through metabolic pathways is termed metabolic flux (Venayak et al., 2015). The flux must be tightly controlled to achieve the three most important characteristics of an industrial strain–yield, titer, and productivity (Venayak et al., 2015; Campbell et al., 2017). These three strategies are being applied to organize the flux of metabolites and to improve host productivity. The first is the rewiring of metabolic flux. The second is the compartmentalization of pathways. The third is the construction of synthetic protein scaffolds.

Rewiring of the metabolic flux can be achieved by deleting genes from the host that, somehow, inhibit the production or accumulation of the desired compound and/or by overexpressing the required pathway enzymes, aiming at the accumulation of a specific product. The advantage of engineering protein scaffolds is the presence of modular protein-protein interaction domains that can be used to adjust the stoichiometry of a given complex in a pathway, thus, allowing the fine-tuning of the metabolic flux (Dueber et al., 2009). With regard to compartmentalization, even though bacteria use the cytoplasm for most of their metabolic reactions, while working with fungal strains, we can take advantage of the numerous subcellular compartments available in their organelles and the cell wall (Hammer and Avalos, 2017). Thus, the metabolic flux toward subcellular compartments can also be engineered to redirect chemical production to beneficial conditions, using several strategies that include the engineering of protein localization tags (Campbell et al., 2017). Another issue that can be overcome by both compartmentalization and engineered protein scaffolds is the loss of intermediates to competing pathways (Wheeldon et al., 2016).

Acetyl-CoA metabolism has been extensively studied, since a number of compounds can be derived from this substrate. These include fatty acids (for biodiesel), polyketides (for antibiotics), and terpenoids, such as ß-carotene and amorphadiene (Yuan and Ching, 2016; Campbell et al., 2017). The significantly regulated central carbon metabolism has been targeted, recently, to increase the metabolic flux toward these metabolites (Campbell et al., 2017; Marella et al., 2018). An interesting design to optimize the use of acetyl-CoA in *S. cerevisiae* cells was adopted by Zhou et al. (2016). The authors constructed a cell chassis in which the fatty acid reactivation pathway was disrupted to stop the inhibition of fatty acid biosynthesis. Subsequently, they built a synthetic chimeric citrate lyase pathway to improve the supply of acetyl-CoA. Using this approach, very high yields of free fatty acids were obtained, with amounts reaching up to 10.4 g/L in a fed-batch fermenter. The free fatty acids were further converted to important oleochemicals, such as alkanes and fatty alcohols. An interesting aspect of the construction of this cell factory is that all constructs were integrated into the genome with no plasmid expression, which is appealing for industrial purposes.

*Y. lipolytica* is a distinctive oleaginous yeast strain whose metabolism is also being widely studied for its high capacity to produce cytosolic acetyl-CoA (Marella et al., 2018). Xu et al. (2016) rewired the central carbon metabolic pathway by overexpressing some alternative routes for the formation of acetyl-CoA, using plasmids containing the ePathBrick technology, a synthetic biology tool that is being used widely in the design of biosynthetic pathways. The authors were able to decouple nitrogen starvation from lipogenesis, enabling the biosynthesis of oleochemicals during the exponential growth phase. Interestingly, overexpression of a protein that exports acetyl-CoA from the mitochondria to the cytosol markedly improved lipid accumulation. Friedlander et al. (2016) further explored the *Y. lipolytica* lipid pathway, by examining when acetyl-CoA is converted to acyl-CoA. The authors overexpressed two heterologous enzymes (DGA1 and DGA2) responsible for the incorporation of acyl-CoA onto the diacylglycerol backbone to synthesize TAGs. This, along with a deletion from a lipase regulator, increased the lipid content to 77%. Both the studies reported the efficiency of using synthetic pathways to increase the production of fatty acids by *Y. lipolytica*, which later can be converted to oleochemicals that are important for industrial applications, including biodiesel, detergents, and bioplastics, among others (Marella et al., 2018).

Another industrially important molecule produced by *Y. lipolytica* is ß-carotene, which is widely used as a color additive and nutritional supplement (Gao et al., 2017). In this study, the authors redirected the carbon flux from acetyl-CoA to ß-carotene by fine-tuning the expression of 11 synthetic genes modulated by strong promoters. For this, a multiple-copy integration strategy was used, and the final product yield was surprisingly higher when compared with the organisms that are commonly used to produce ß-carotene industrially.

**Figure 3:**
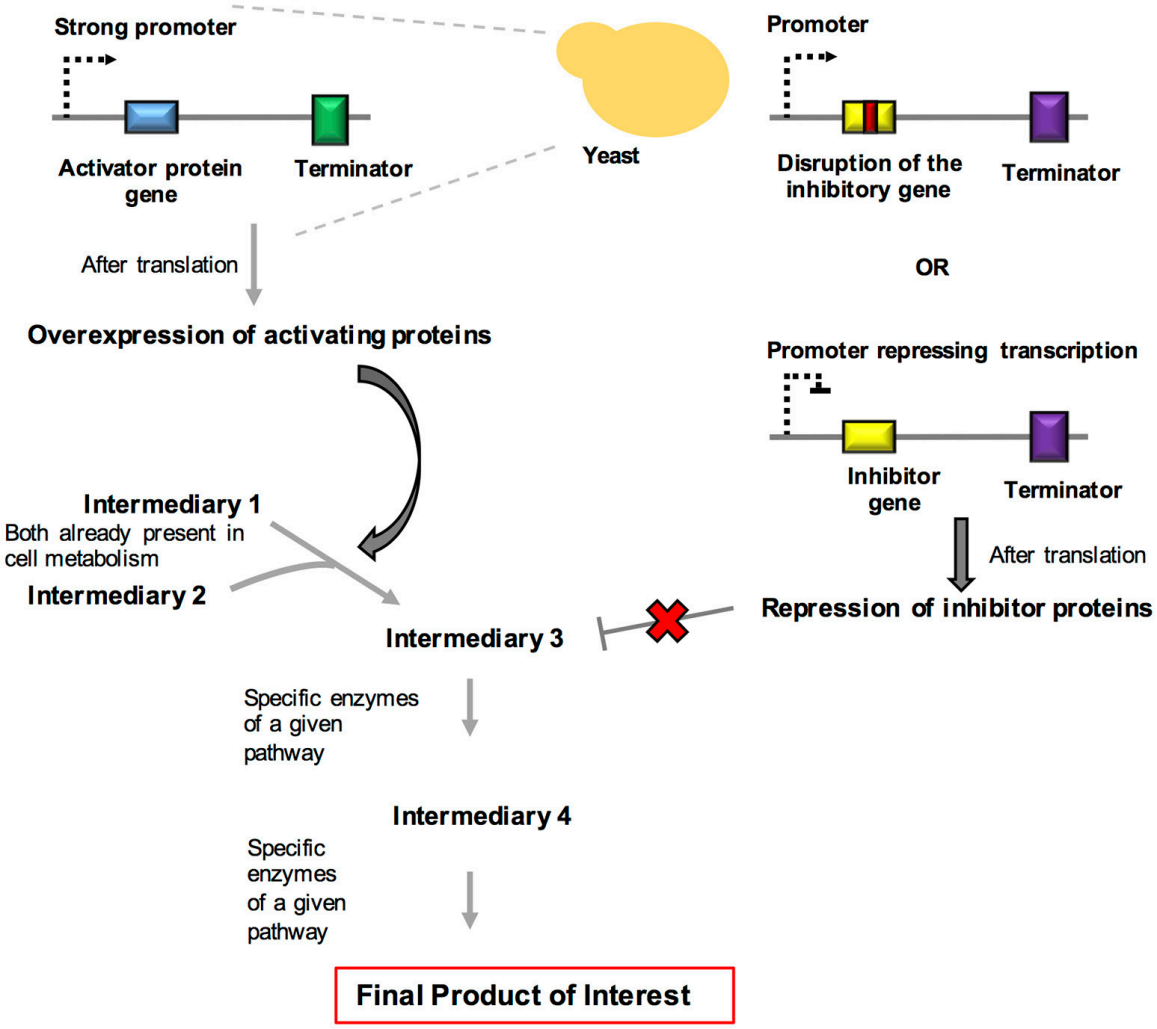
Schematic representation of systems and synthetic biology approaches used for rewiring and for the dynamic control of metabolism. In this strategy, we summarize the overexpression of activating proteins and the repression of inhibitory proteins in order to propitiate a redesign of metabolites generation in yeast.

*R. toruloides* is a yeast strain that is growing in importance in the field of metabolic engineering. Remarkable results have been obtained in the production of fine chemicals through the process of degradation of complex substrates, such as xylose and aromatics, derived from lignin. Yaegashi et al. (2017) reported the high-level production of two terpenes by *R. toruloides*. Bisabolene is the precursor of bisabolane, which is a biosynthetic alternative to D2 diesel. Amorphadiene is the precursor of the antimalarial drug artemisinin. The production of these compounds was achieved by taking advantage of the large amounts of acetyl-CoA present in the cytosol of this organism and by expressing codon-optimized versions of the genes encoding bisabolene synthase (*bis*) and amorphadiene synthase (*ads)*. Remarkably, the production of these chemicals increased when the organism grew using lignocellulose hydrolysates when compared with to purified substrates. A brief overview about the systems and synthetic biology strategies used for rewiring and dynamic control of metabolism in fungi are summarized in Figure 3.

## Dynamic control through genetic circuits

Even after considering the aforementioned accomplishments, it is important to note that there is still a huge difference between static and dynamic metabolism. Biological systems are much more complex than a physical, steady system. Results are hard to reproduce and, thus, hard to predict. This is especially true if we only consider a limited part of the system, such as enzymes in a metabolic pathway (Chubukov et al., 2016). Those components are not static, as their levels are always in response to changes in cellular environment. Synthetic biology can explore the fine-tuning of key metabolic steps through genetic circuits, which can optimize cell factories and bypass the any given specific limitation. This can also be seen under the light of a systems biology approach, not only for coupling chemical production with growth but also because one can use stoichiometric modeling to help in host engineering (Venayak et al., 2015; Chubukov et al., 2016; Teixeira et al., 2017).

One constraint that can be circumvented by the use of genetic circuits is the rate of fungal biomass production vs. the amount of the desired products generated (and also vs. all undesired by-products). An example of a study that took advantage of genetic circuits was performed by Williams et al. (2015). The authors constructed an ON-OFF circuit to overcome the metabolic burden by separating growth and compound production. This system was based on quorum sensing, using a pheromone and RNAi methodology. In this system, genes related to the production of the compound of interest were kept OFF, while genes related to growth were ON. When the population reached an optimal growth, genes related to biomass production were knocked down by RNAi while those related to the metabolic pathway of interest were activated. The authors used this strategy to increase the yield of *p*-hydroxybenzoic acid (PHDA) in *S. cerevisiae* and reported the highest PHDA yield ever achieved in yeast. Still, in this sense, ON-OFF genetic circuits can also be achieved by adding responses to temperature or other inducers, such as isopropyl β-D-1-thiogalactopyranoside (Venayak et al., 2015).

Continuous genetic circuits can also be constructed for the dynamic control of target metabolic pathways, coupling gene expression to the sensing of a specific metabolite. Metabolite sensors allow the circuit to respond in accordance with the cellular environment so that they become sensitive to variations. As an example, Xu et al. (2014) developed a continuous circuit triggered by malonyl-CoA concentrations. The promoters that were activated by malonyl-CoA induced a consumption pathway, while the same substrate repressed a production pathway. Thus, this intermediate compound was used to regulate the entire fatty acid biosynthetic pathway.

Another strategy for the dynamic control of metabolic pathways was employed by Teixeira et al. (2017). In this case, the authors studied the production of fatty alcohols from free fatty acids. For this, they dynamically expressed the fatty acyl-CoA synthase gene *faa1* under the control of different promoters to prevent the accumulation of free fatty acids in industrial mutant strains, thus avoiding the loss of precursors to the extracellular medium. This approach enhanced the production of fatty alcohols and expanded the knowledge regarding the control of metabolite flux of this pathway. This is an excellent example of the use of dynamic control to increase industrial production. Venayak et al. (2015) supplied other examples to explain all the benefits and drawbacks of this approach. Algorithms for stoichiometric metabolic models are currently available to help with the understanding of how cell network contributes to the yield of the final product. Some examples are OptStrain and OptForce, which can be used to find additional reactions that can be targeted and to identify the pathways that need to be engineered. In this sense, these algorisms can be applied to guide host engineering approaches and to enhance the benefits of building genetic circuits (Chubukov et al., 2016). In a generic manner, dynamic regulatory circuits can be combined with all the previously cited tools and strategies to provide a more refined and productive mutant fungal strain to address the biotechnological demand in industrial processes.

## Synthetic biology applied to secondary metabolism

**Figure 4:**
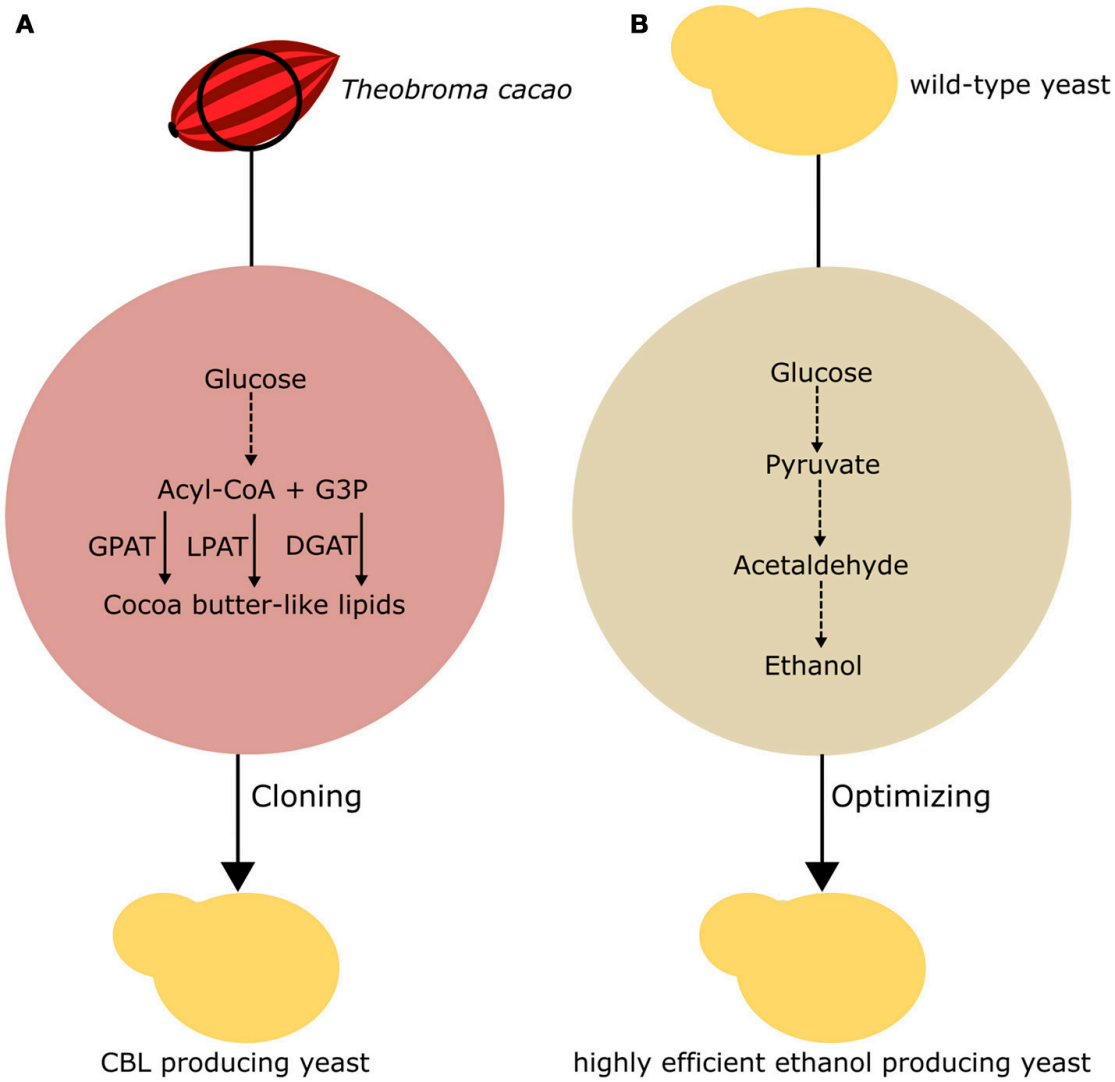
Basic approaches to chemical production in fungi. **(A)** Implementation of a CBL pathway in yeast. G3P, glycerol-3-phosphate; GPAT, glycerol-3- phosphate acyltransferase; LPAT, lysophosphatidic acid acyltransferase; DGAT, acyl-CoA:diacylglycerol acyltransferase **(B)** Optimization of existing fermentation pathways in yeast to enhance first-generation ethanol production.

In the last 15 years, the research community has used two approaches to produce fine chemicals in fungi. One approach is the implementation of new metabolic pathways through recombinant DNA techniques. The second approach is the engineering of existing pathways to enhance the yield and purity of existing metabolites. While in this review, we focus on the production of fine chemicals, molecules with low aggregated value and high-volume production have been a point of interest in of recent studies, as summarized in Figure 4.

The bioproduction of lipid molecules in fungi can provide novel renewable and sustainable material for the production of food ingredients independent of plant cultivation, climate changes, and seasonal availability. Lipids extracted from plants, such as the cocoa tree (*Theobroma cacao*), can be utilized for industrial purposes. The lipids extracted from *T. cacao* beans are the basic component of cocoa butter, a valuable ingredient that is becoming increasingly scarce in the market. In this sense, Wei et al. (2018) reported the application of rational metabolic engineering to produce a CBL product in yeast. To obtain a strain producing a CBL product, the authors precisely determined the genes that are responsible for the production of the TAGs that compose cocoa butter, so that these genes could be functionally expressed in *S. cerevisiae*. The main composition of this triacylglycerol product is 1,3-dipalmitoyl-2-oleoylglycerol, 1-palmitoyl-3-stearoyl-2-oleoylglycerol, and 1,3-distearoyl-2-oleoylglycerol. These TAGs fail to naturally accumulate in large amounts in *S. cerevisiae*. Wei et al. (2018) proposed that the enzymes responsible for the synthesis of TAGs from *T. cacao* could be cloned into *S. cerevisiae*, favoring the production of the adequate TAG to obtain the (CBL) product.

To investigate this hypothesis, the authors cloned cocoa glycerol-3-phosphate acyltransferase, lysophospholipid acyltransferase, and diacylglycerol acyltransferase in *S. cerevisiae* through Gibson assembly into the pBS01A plasmid (Wei et al., 2018). Interestingly, some of those genes were amplified from cocoa cDNA and showed different sequences from the previously annotated genes. Additionally, a valuable use of this approach is the possibility of directly characterizing and testing the activity of plant genes in yeast. As a means to optimize the production of CBL, the authors combined the expression of the cocoa genes with two previously reported genes for the production of CBL compound (Wei et al., 2017b). The resulting strains were able to produce significantly higher amounts of the TAGs of interest than the control strain, with an increased total fatty acid production up to 84%.

### Alkaloids

Alkaloids play a significant role in human health, and these are structurally and functionally diverse molecules. Their health applications are diverse, ranging from the treatment of pain (opioids) to cancer (vinblastine and vincristine). Owing to their structural complexity, most of those important chemicals are produced through extraction or semi-synthesis from plant species. Addressing problems like seasonal variations in crop yields and batch variation of *Papaver somniferum*, a plant that produces morphine and other opioids, Galanie et al. (2015) developed a proof-of-concept synthetic circuit composed of more than 20 genes that can produce opioids in *S. cerevisiae*. Similar advances were made by Li and Smolke (2016) for the production of noscapine, an anticancer drug isolated from *P. somniferum*.

The assembly and production of these molecules in yeast allowed the establishment of new sources for these key molecules. They also enable researchers to use yeast as a platform for enzyme engineering, to generate new tools that can be used in drug discovery. The study conducted by Li and Smolke (2016) also raised important concerns regarding the safety and ethics of producing psychoactive drugs in an easy manner in organisms such as *S. cerevisiae*. Although the yield obtained by Galanie et al. (2015) was low (which makes licit and illicit applications of this technology unlikely), advances in the yeast biosynthesis of other non-alkaloid psychoactive compounds such as Δ9-tetrahydrocanabinolic acid (THCA), a precursor of the main psychoactive *Cannabis* constituent (tetrahydrocannabinol (THC)) (Zirpel et al., 2015), has raised concerns about the safety of microbial production of addictive active pharmaceutical ingredients.

### Nonribosomal peptides

The production of nonribosomal peptides (NRP) is of interest for their use in synthetic biology approaches, since the modularity of NRP synthetases (NRPSs) allows enhanced compound production or the synthesis of entirely new structures. A milestone in the application of synthetic biology to produce NRP using *S. cerevisiae* was reported by Awan et al. (2017). They managed to express the whole biosynthetic pathway of benzylpenicillin in this yeast, thus, validating a screening method for antibiotic NRP production. The benzylpenicillin biosynthetic pathway consists of five enzymes (encoded by *pcbAB, npgA, pcbC, pclA*, and *penDE*). *pcbAB* and *npgA* are responsible for the synthesis of the benzylpenicillin (PEN) precursor, amino-adipyl-cysteine-valine (ACV), while the products of *pcbC, pclA*, and *penDE* are responsible for the conversion of ACV to PEN.

Although the heterologous production of ACV in *S. cerevisiae* and of PEN in *Hansenula polymorpha* were previously reported by (Gidijala et al., 2009; Siewers et al., 2009), the approach used to express the whole PEN pathway in *S. cerevisiae* was a simple, inexpensive, yet, powerful platform to screen relevant NRP pathways. This approach enabled the development of new NRP through the combinatorial assembly of NRPS pathways and construction of chimeras (Awan et al., 2017).

### Flavonoids

Flavonoids are a class of polyphenolic compounds produced by plants. They have a variety of uses in modern and traditional health practices, with benefits including antimicrobial and antioxidant activities (Marín et al., 2015; Skrovankova et al., 2015). Breviscapine is a flavonoid extract that is used in Chinese medicine. This flavonoid is composed mainly of two molecules, scutellarin and apigenin 7-O-glucuronide. It is obtained through the extraction of vegetal tissues from *Erigeron breviscapus*, which has resulted in scarce supply as its popularity has increased over the past 30 years. Therefore, new methodologies to produce the active molecule are needed to ensure the supply of this active pharmaceutical ingredient. In an attempt to produce the main components of breviscapine, Liu X. et al. (2018) identified and expressed components of the breviscapine pathway in yeast, and they were able to produce the flavonoids scutellarin and apigenin 7-O-glucuronide from glucose. Their success exemplifies the potential of synthetic biology as a metabolic pathway elucidation tool, which, in this example, aided the researchers to elucidate the breviscapine biosynthetic pathway. This example shows how synthetic biology can have a huge impact on the biosynthesis of flavonoids, therefore, establishing a constant supply chain of such natural products through fungal bioproduction.

### Glycosides

The burgeoning prevalence of metabolic syndrome, obesity, and diabetes is increasing the need for alternatives to sugar. Steviosides are safe sweeteners that are extracted from the leaves of *Stevia rebaudiana*. They are glucosides composed of diterpenoids that are covalently bonded to three glucose molecules. A collateral effect of those sweetener molecules is a bitter off-flavor. In this sense, researchers from the biotechnology company Evolva (Olsson et al., 2016) developed strains of *S. cerevisiae* that produce novel next-generation stevioside with a reduced bitter taste. Through homology modeling and identification of key target amino acids present in the glucosyltransferase UGT76G1, the authors were able to obtain *S. cerevisiae* strains with increased accumulation of rebaudioside D and M steviosides with a less bitter taste and enhanced sweetness. Their success indicates the potential of this approach for yeast metabolic engineering of sugar replacements.

## Eukaryotic promoters and transcription factors: the blocks to control gene expression

The role of TFs and TF motifs can directly or indirectly drive transcription. These proteins control transcription by transforming physiological and environmental signals into patterns of gene expression (Weingarten-Gabbay and Segal, 2014), thus, acting as biosensors to turn transcription ON or OFF (D'Ambrosio and Jensen, 2017). Transcription factors recognize specific sequences in the DNA, collectively abbreviated as TFBSs. With the aid of computational approaches, TFBSs can be represented as position weight matrices (PWMs) in an attempt to represent the statistical or binding energy of the DNA-protein interaction depending on the data type that originated from the PWM (Schipper and Gordân, 2016). Additionally, more comprehensive models that include Bayesian networks or support vector machine based models have been reported recently, which are reviewed in Boeva (2016).

The discovery of new TF motifs is a step forward in understanding gene regulation, which can serve as a basis for new bioengineering applications. However, finding TFBS motifs is a difficult task, since primary nucleotide sequence is not the unique characteristic that specifies a TF target. Additional factors, such as multiple modes of DNA binding, DNA modification, DNA shape, genomic context, and coding and noncoding (genetic) variation can change TF nucleotide sequence preferences, as reviewed in Inukai et al. (2017) and Siggers and Gordân (2014).

Studies using high-throughput data regarding TF binding specifity are another source of valuable information on parameters affecting gene regulators. In this sense, Gordân et al. (2011) suggested that several TFs of *S. cerevisiae* have a primary and secondary binding motif, which may even perform distinct regulatory functions. These findings indicate a property that could be further explored in motif search algorithms that focus on yeasts. Even with many experimental approaches that generate high-throughput data, it is impossible to test all the environmental conditions that a natural regulatory network is able to respond to. Despite this limitation, several methods and approaches have been used to identify motifs in high-throughput data. In this regard, computational motif discovery tools that deal with large data (like those generated from ChIP-seq, SELEX, or ChIP-chip assays) that are currently being used include HMS (Hu et al., 2010), cERMIT (Georgiev et al., 2010), HOMER (Heinz et al., 2010), diChIPMunk (Kulakovskiy et al., 2013), MEME-ChIP (Machanick and Bailey, 2011), rGADEM (Mercier et al., 2011), POSMO (Ma et al., 2012), XXmotif (Hartmann et al., 2013), FMotif (Jia et al., 2014), Dimont (Grau et al., 2013), and DeepBind (Alipanahi et al., 2015). Particularly, rGADEM, HOMER, POSMO, Dimont, and ChIPMunk are tools with good performance (Boeva, 2016; Jayaram et al., 2016).

### Promoters

Bioinformatics research is being allied with synthetic biology for the discovery of new modules and their interactions. Yeast promoters have motifs/sequences that are necessary for promoter function and for assembly of the preinitiation complex, which leads to the recruitment of the entire cellular transcription machinery (Thomas and Chiang, 2006). The core elements in yeast promoters are nucleosome-free regions located approximately 140 base pairs (bp) at the beginning and at the end of the genes that are rich in adenine (A) or thymine (T). Additionally, transcription start sites (TSSs), TATA-box, and upstream activation sites located several hundred bp upstream of the TSS are required. Analogously, upstream repressing sequences can also be present in natural promoters (Venters and Pugh, 2009; Sesma and von der Haar, 2014).

Promoter prediction is a hurdle. Transcriptional initiation is the first step in gene expression and, so, is an important control point. Despite its importance, eukaryotic promoter prediction is not simple because of the structural complexity of natural *cis*-regulatory elements (Pedersen et al., 1999; Yella and Bansal, 2017). In the past years, promoter prediction tools have improved, which have been driven by new high-throughput data generated by next-generation sequencing and by the application of analytical machine learning methods, such as the support vector machine, neural networks, and naïve Bayesian classifier (Singh et al., 2015). However, until now, no precise prediction tools are available. Yet, several software use eukaryotic promoter characteristics that are specific to a given specie or to animals, which are tools that work with a specific methodology and precision.

Owing to the complexity of promoter prediction in general, we presents prediction below some useful tools that have been applied in fungi/yeast or that could be adapted to these organisms. For example, Shahmuradov et al. (2017) attained better performance than previous programs by using a novel prediction tool for plant promoters, which was named TSSPlant. This tool was created by using large promoter collections from plant promoter databases to identify most relevant promoter *cis*-regulatory elements. This was followed by the use of a neural network with back propagation to create a promoter classifier, thus, allowing an improvement in accuracy. Another notable approach to solve the prediction bottleneck was described by Umarov and Solovyev (2017). The authors developed a general method for the recognition of promoters by constructing a predictive model with convolutional neural networks. To test the method and to prove its universality, the authors used promoter sequences from distinct organisms (bacteria, human, mouse, and plant). For each organism, the best accuracy was achieved when it was compared with the existing tools.

**Figure 5:**
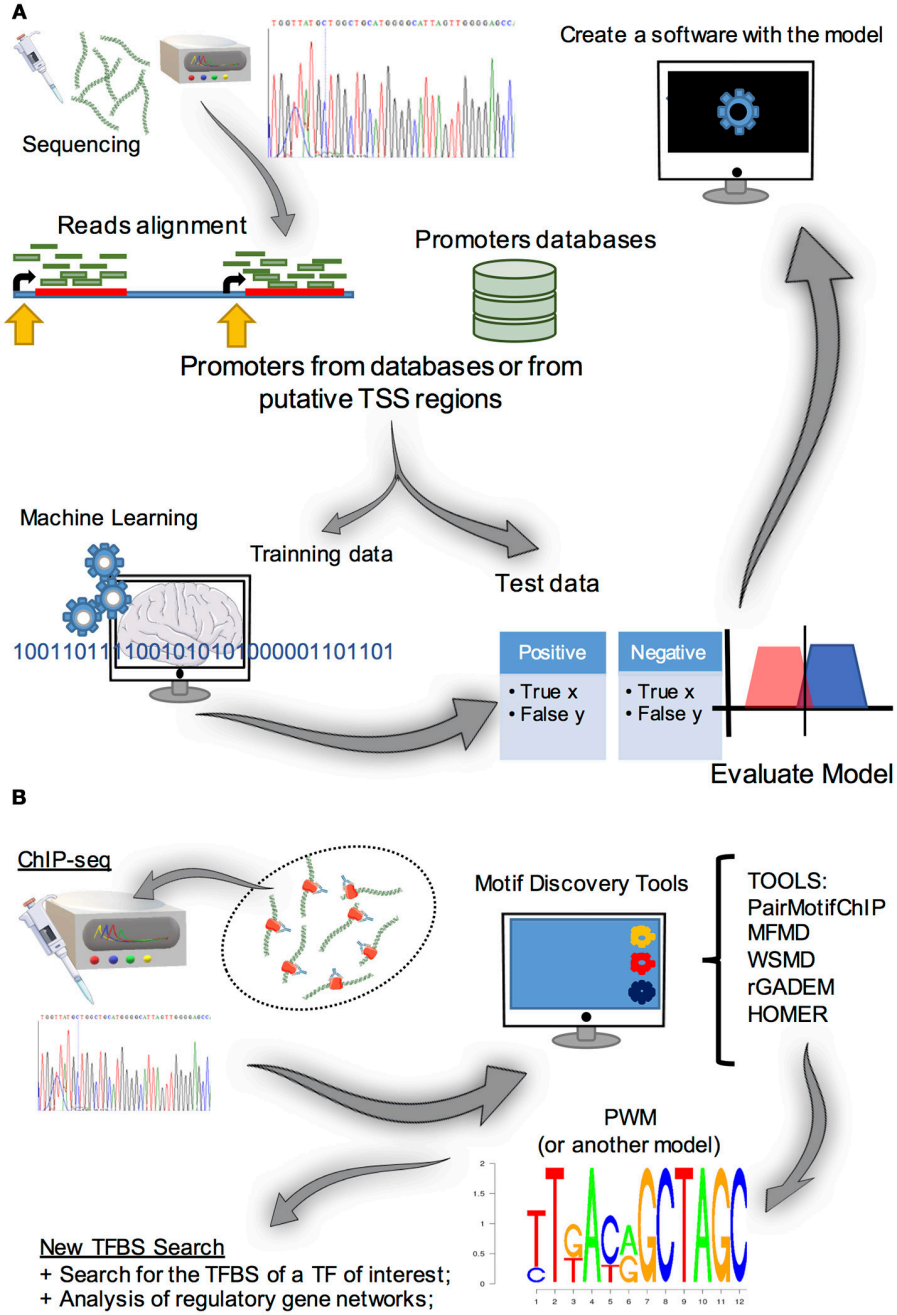
**(A)** Summary of the approaches used for promoter prediction. The first step is to obtain a large amount of promoter data (obtained from promoter databases or by analyzing RNA-seq 5′ cap transcripts and obtaining putative TSS, for example). Subsequently, methods to find patterns in data (e.g., machine learning algorithms) are used in a set of the initial data. After that, the model created must be evaluated by classifying test data set, which is a small portion of the initial data, to evaluate its predictive power. Once the model is obtained, it can integrate a promoter predictive tool. **(B)** Motif discovery strategy. Basically, to find *de novo* TFBS motifs, experimental procedures and computational analysis are required. Initially, a ChIP-seq (or a SELEX or a ChIP-chip) experiment is run to obtain sequences that contain the TFBS of interest. As these sequences are usually larger than the motifs, it is necessary to use a software in these data to reveal possible TFBS motifs and represent them as PWM (or another model), for further gene transcription regulation studies.

In addition to the aforementioned prediction methods that deal with pre existing data, several studies have used transcriptomics to find putative TSSs. With specific RNA-seq protocols to obtain 5′ regions (Gowda et al., 2007; de Hoon and Hayashizaki, 2008), the reads can be compared with the available genome annotation data. Analysis of nearby regions could then be performed using alignment and conventional motif discovery tools (discussed in detail below). This approach provided useful information about the core promoters of *Schizosaccharomyces pombe* and *A. nidulans* (Sibthorp et al., 2013; Li et al., 2015). These studies highlight a relevant experimental/computational strategy to obtain data regarding regulatory elements in fungal promoters. Yet, since this study, no recent reports about fungal promoter prediction tools have been published. Hence, we suggest that these aforementioned notable approaches could be integrated to aid the creation of new software and new pipelines of promoter discovery in these biotechnologically relevant organisms (Figure 5).

### Bioinformatic approaches for the identification of TFBS

Considering current efforts in analyzing large amounts of data, Yu et al. (2016) proposed a new algorithm, PairMotifChIP, for this purpose. This tool can identify motifs by extracting combining pairs of an “l” width in the input sequences that have small Hamming distance, distinguishing the motifs from random overrepresented sequences by probabilistic analysis and then combines the remaining sequences to form motifs. This tool runs very fast and does not require previous user information (Yu et al., 2016). Caldonazzo Garbelini et al. (2018) created a new approach for motif discovery by making use of a genetic algorithm to escape from optimal local solutions. The algorithm was designated as Memetic Framework for Motif Discovery (MFMD). The study made use of a version of the semi-greedy heuristic to build initial solution population and genetic algorithms (as a global optimizer) to develop these initial solutions (Caldonazzo Garbelini et al., 2018). Another tool that was also built to escape from optimal local solutions is weakly-supervised motif discovery (WSMD). The WSMD algorithm uses a latent support vector machine optimization strategy and learns PWM in a continuous space, which reduces the loss of information. Thus, the quality of the motifs is improved (Zhang et al., 2017). All of these studies feature a comparative analysis with the currently used motif discovery tools and seem to have better performance.

While these tools may help in studies to overcome limitations in motif discovery, working with high-throughput data remains a bottleneck. To deal with this, Guzman and D'Orso (2017) developed CIPHER, a framework that integrates complex bioinformatics tools to analyze different types of next-generation sequencing data (e.g., ChIP-seq, RNA-seq, DNase-seq). This tool provides several types of analyses that include differential gene expression, peak annotation, and reasoning (with HOMER) in an easy-to-use way. This tool also allows parallelization of processing for the better use of local hardware, as well as a quality control module to identify possible errors, contaminations, and bias in input sequences, which generates accurate results (Guzman and D'Orso, 2017).

## Conclusions

Systems and synthetic biology are playing a pivotal role in the development of tools for engineering yeast and filamentous fungi. Studies based on them have shed light on how single modules can influence an entire and robust system. Besides, the particularity of each tool presented in this review, the approaches to engineer fungi as cell factories for biotechnological industrial processes, can be successfully combined to guarantee a viable and cost-efficient strategy. The tools described here can cover a wide area of applications, substantially improving the already existing methodologies to engineer these organisms. Still, despite the great promises of synthetic biology, the genetic manipulation of filamentous fungi remains to be one of the major challenges for industrial applications. The difficulty of manipulating their genomes still hinders the use of metabolic engineering for filamentous species. However, synthetic biology approaches used for yeast are significantly contributing to the spread of methodologies and tools for engineering microbes to produce high value-added products at an achievable cost and to benefit equipoise. In general, synthetic biology approaches have presented successful examples of management and the acquisition of mutant strains that meet industrial demand. Finally, we anticipate that novel computational tools, especially for the investigation and design of regulatory elements, will play a pivotal role in future engineering attempts of these remarkable organisms.

## Author contributions

LM-S and RS-R conceived the work. LM-S, LN, AS-M, GL, MC, and RS-R assembled the first draft of the manuscript. LM-S and RS-R revised the final version of the work. All the authors have read and approved the final version of the manuscript.

### Conflict of interest statement

The authors declare that the research was conducted in the absence of any commercial or financial relationships that could be construed as a potential conflict of interest. The reviewer EB and handling Editor declared their shared affiliation.
